# The Role of NLRP3 Inflammasome in Radiation-Induced Cardiovascular Injury

**DOI:** 10.3389/fcell.2020.00140

**Published:** 2020-03-12

**Authors:** Shanshan Huang, Jing Che, Qian Chu, Peng Zhang

**Affiliations:** ^1^Department of Oncology, Tongji Hospital, Tongji Medical College, Huazhong University of Science and Technology, Wuhan, China; ^2^College of Life Sciences, Wuhan University, Wuhan, China

**Keywords:** NLRP inflammasome, radiation, cardiovascular injury, atherosclerosis, radiation fibrosis

## Abstract

The increasing risk of long-term adverse effects from radiotherapy on the cardiovascular structure is receiving increasing attention. However, the mechanisms underlying this increased risk remain poorly understood. Recently, the nucleotide-binding domain and leucine-rich-repeat-containing family pyrin 3 (NLRP3) inflammasome was suggested to play a critical role in radiation-induced cardiovascular injury. However, the relationship between ionizing radiation and the NLRP3 inflammasome in acute and chronic inflammation is complex. We reviewed literature detailing pathological changes and molecular mechanisms associated with radiation-induced damage to the cardiovascular structure, with a specific focus on NLRP3 inflammasome-related cardiovascular diseases. We also summarized possible therapeutic strategies for the prevention of radiation-induced heart disease (RIHD).

## Introduction

Chronic health problems in cancer survivors include cardiovascular diseases (CVDs), which are the primary causes of morbidity and mortality in this population. There is a significant correlation between increased risk of CVDs and chest irradiation, which results in short-term and long-term cardiovascular complications in cancer survivors (Abe et al., 2016). Although acute pericarditis can result from high dose radiation, radiation-induced heart injury may take decades to become symptomatic (Nielsen et al., 2017). Common CVDs include accelerated atherosclerosis, myocardial remodeling, fibrosis, and injury to cardiac valves. Recent epidemiological, clinical, and preclinical studies have shown strong evidence that ionizing radiation can cause cardiovascular injury (Stewart et al., 2013; Yusuf et al., 2017; Wang H. et al., 2019). The underlying mechanisms of radiation-induced cardiovascular injury have not been characterized due to the wide spectrum of complex effects of ionizing radiation on the cardiovascular system.

Recent studies showed that NLRP3 inflammasome upregulation and activation were associated with radiation damage (Wei et al., 2019). However, the role of the NLRP3 inflammasome in radiation-induced damage requires further characterization. In this review, we focused on relevant epidemiological and clinical evidence for radiation-induced CVDs. We also reviewed the role of the NLRP3 inflammasome in radiation-induced injury, and highlighted possible therapeutic strategies for preventing radiation-induced heart disease (RIHD).

## Overview of Radiation-Induced Cardiovascular Diseases

### Clinical Evidence for Radiation-Induced Cardiovascular Diseases

Survivors of childhood cancer and Hodgkin’s lymphoma who experienced conventional fractionated radiotherapy (total tumor dose of 30–40 Gy) are at greater risk of late cardiac death than the general population (Stewart et al., 2013). Long-term follow-up of over 1,400 patients with Hodgkin’s lymphoma with a history of radiotherapy showed a significant increase in standardized incidence ratio of myocardial infarction (MI), angina, valve disease, and congestive heart failure. In addition, patients who received radiotherapy at a young age (<20 years-old) are at significantly higher risk for MI (5.4 standard incidence ratio) than patients irradiated at age 36–40 (2.6 standard incidence ratio). Anthracycline chemotherapy further increased the risk of cardiovascular events in young patients with a history of thoracic irradiation (Aleman et al., 2007). Head and neck cancer patients with a radical radiotherapy history often experience a significant dose of radiation to the carotid arteries. Subsequent vascular injury may result in atherosclerosis, and increased risk of stroke and transient ischemic attack (TIA) (Scott et al., 2009).

Mulrooney et al. (2009) and Tukenova et al. (2010) showed a relationship between cardiovascular death and cardiac radiation dose in childhood cancer survivors. These studies showed that the relative risk of cardiovascular death caused by radiotherapy was significantly associated with mean heart dose, particularly when cardiac doses were ≥ 15 Gy. Mean heart doses over 35 Gy were linked with even greater hazard ratios. Prospective studies showed that the majority of survivors of Hodgkin’s lymphoma exhibited asymptomatic cardiovascular abnormalities (e.g., conduction defects, valve dysfunction, and diastolic dysfunction). These findings suggested that occult RIHD may be underestimated in this patient population. Whether low-dose irradiation damages the cardiovascular system is unknown.

Mean heart doses and dose distribution to certain cardiac structures were estimated in patients with breast cancer who underwent post-operative irradiation (50 Gy in 25 fractions) up to the early 1990s (Taylor et al., 2007). Left-side breast cancer patients, of whom some received internal mammary chain radiation, experienced total average doses of 3–17 Gy across the entire heart. A large-scale investigation suggested that exposure of the internal mammary chain to radiation correlated with a significantly increased risk of cardiovascular abnormalities (average cardiac dose was 6–15 Gy). Patients who underwent breast irradiation alone with a mean heart dose < 7 Gy were not at increased risk for development of CVD (Hooning et al., 2007). However, the effect of low dose irradiation (<2.5 Gy) on the cardiovascular system is unknown. A life-span investigation of Japanese atomic bomb survivors indicated that cardiac abnormalities such as inflammation and fibrosis were increased by exposure to low dose radiation (Shimizu et al., 2010).

### Experimental Evidence for Radiation-Induced Cardiovascular Diseases

Use of experimental animals to estimate RIHD is convenient due to shorter life spans and ease of follow-up. Radiation-induced cardiovascular injury depends on timing, dose and fraction, and extent of radiation exposure to the heart and blood vessels. However, most animal studies have applied single high doses or a total high dose across a limited number of fractions (Sylvester et al., 2018). Local single high doses of radiation are more frequently applied to young adult (male) animals. Rodent models are widely used to identify potential biological mechanisms of radiation-induced cardiac injury. Larger animals, such as rats, rabbits, and cynomolgus monkeys, have been used for decades to study RIHD (Boerma, 2012).

Although myocardial changes have been shown to be similar in animals and humans, radiation-induced accelerated atherosclerosis has not been frequently observed in common animal models (Boerma et al., 2016). Several research groups have reported that increased perivascular and interstitial collagen deposition in the myocardium was observed several months after local cardiac radiation in mice (Schultz-Hector, 1992; Unthank et al., 2015). Increased collagen deposition was accompanied by local increased fibrotic cytokine expression. Boerma et al. (2013) found that induction of TGF-β expression further enhanced radiation-induced cardiac fibrosis. Moreover, this radiation-induced fibrosis worsened with loss of microvasculature density and function in the myocardium (Taunk et al., 2015).

To investigate radiation-induced accelerated atherosclerosis, inclusion of additional cardiovascular risk factors in experimental models is necessary. Genetically modified mouse models susceptible to atherogenesis, such as LDL receptor-deficient mice (*Ldlr*^–/–^) and apolipoprotein E-deficient mice (*Apoe*^–/–^), are commonly used (Schiller et al., 2001; Stewart et al., 2006). High dose radiation to the neck of *Apoe*^–/–^ mice led to increased atherosclerotic plaque formation and increased macrophage abundance in the carotid artery (Hoving et al., 2008). In addition, local heart radiation resulted in microvascular injury and atherosclerosis in the coronary arteries of *Apoe*^–/–^ mice that was more extensive (Gabriels et al., 2012). Pathophysiological changes in the cardiovascular systems of small animals exposed to radiation are similar to those in clinical specimens, including increased intima-media thickness and more artery wall lesions (Unthank et al., 2019).

### Pathological Features of Radiation-Induced Cardiovascular Diseases

Atherosclerosis and fibrosis are typical pathological changes in RIHD, but the pathophysiology of these changes is poorly understood (Yarnold and Brotons, 2010). Many of the pathological changes in vascular structure secondary to radiation may be much the same as those observed in typical atherosclerosis. However, radiation-accelerated coronary atherosclerosis results in more extensive media damage and adventitia fibrosis compared with ordinary coronary atherosclerosis. In addition, more lipid and calcium deposition has been shown to occur in radiation-induced fibrous tissue of intimal plaques than in non-irradiated atherosclerotic plaques (Virmani et al., 1999). Brosius et al. (1981) found that more than one-fourth of the major coronary arteries were nearly completely narrowed when cross-sections were evaluated. The proximal half of the right and left anterior descending coronary arteries are frequently damaged by radiation (Brosius et al., 1981). Moreover, intramyocardial coronary thickening and myocardial fibrosis of the right ventricle have been observed in response to radiation. The endocardium was significantly thickened in the right ventricle of many patients who underwent radiation (Tadic et al., 2017). Similarly, valve thickening, particularly of the tricuspid valve, occurred in over 80% of patients who underwent irradiation (Gujral et al., 2016a). Nearly all patients experienced pericardial and epicardial fibrosis following radiotherapy, and pericardial tamponade was also common.

Cervical radiotherapy-induced carotid lesions exhibit a greater degree of stenosis than non-typical atherosclerotic lesions. Radiation-induced carotid lesions are frequently observed in the external and common carotid artery. Similar to radiation-accelerated coronary atherosclerosis, ionizing irradiation-induced carotid stenosis presents a more stable phenotype than *de novo* atherosclerotic plaques (Fokkema et al., 2012). Macrophage infiltration was observed less frequently in response to radiation, but more fibrous plaques were present following cervical irradiation-induced carotid lesions (Gujral et al., 2016b).

## Radiation and NLRP3 Inflammasome Activation

### Overview of the NLRP3 Inflammasome

A sensor protein (NLRP3), an adaptor protein (apoptosis-associated speck-like protein containing a caspase recruitment domain, ASC), and an effector protein (cysteinyl aspartate specific protease 1, caspase-1) comprise the NLRP3 inflam- masome. Nucleotide-binding domain and leucine-rich-repeat-containing family pyrin 3 is comprised of three domains: an amino-terminal pyrin domain (PYD), a central NATCH domain (an evolutionarily conserved protein domain contains NLP family apoptosis inhibitor protein, MHC class II transcription activator, incompatibility locus protein from *Podospora anserina*, and telomerase-associated protein), and a carboxy-terminal leucine-rich repeat (LRR) domain (Schroder and Tschopp, 2010). The NATCH domain possesses ATPase activity responsible for NLRP3 self-assembly and function. The LRR domain is responsible for self-inhibition through the NATCH domain. Apoptosis-associated speck-like protein containing CARD (ASC) functions as an adaptor protein with two protein-interacting domains. The amino-terminal domain of the ASC is PYD, and the carboxy-terminal domain is the caspase recruitment domain (CARD) that interacts with inflammatory caspase-1. Upon stimulation, oligomerization of NLRP3 occurs via NATCH–NATCH domain interactions, following by the recruitment of ASC and caspase-1 via homotypic interactions with CARD. Activated caspase-1 then cleaves the pro-inflammatory cytokines IL-1β and IL-18 to their bioactive forms (Swanson et al., 2019). In addition, inflammasome activation results in pyroptosis, a form of cell death characterized by loss of cell integrity, cellular swelling, and lysis (Yuan et al., 2018). Pyroptosis correlates with active IL-1β and IL-18 release by three different mechanisms: secretion through pores formed by active gasdermin D (GSDMD) on the membrane, shedding of microvesicles, and lysosome-mediated exocytosis (Semino et al., 2018). Endogenous danger-associated molecular patterns (DAMPs), such as high mobility group box 1 (HMGB1), APT, and IL-1α produced by stressed cells further activate the NLRP3 inflammasome. Activation of these signaling pathways results in an enhanced inflammatory response (Baldrighi et al., 2017).

In addition to the role of canonical NLRP3 function in activation of caspase-1, non-canonical activation of NLRP3 activated caspase-4/5 in humans and caspase-11 in mice in a TLR-independent manner (Vigano et al., 2015; Yi, 2018). A recent study suggested that NIMA-related kinase 7 (NEK7) might be critical to activation of the NLRP3 inflammasome. Upon inflammasome activation, NEK7 was shown to oligomerize with NLRP3 to form a complex, which indicated that NEK7 was a component of the NLRP3 inflammasome (He Y. et al., 2016).

### Radiation Triggers NLRP3 Inflammasome Activation

Inflammasome activation is considered a two-step “priming and activation” process. Priming is characterized by upregulation of the expression of inflammasome core components and induction of post-translational modifications (PTMs) such as ubiquitylation, SUMOylation, and phosphorylation (Swanson et al., 2019). In addition, interfering RNAs modulate the expression of NLRP3 at the transcriptional level (Tezcan et al., 2019). The second step initiates inflammasome formation upon activation. Nucleotide-binding domain and leucine-rich-repeat-containing family pyrin 3 is an effector of the innate immune response that recognizes pathogen-associated molecular patterns (PAMPs) or DAMPs by engaging pattern recognition receptors (PRRs) (Kelley et al., 2019). In addition, NLRP3 can be activated by a number of other stimuli. Furthermore, non-infectious stimuli such as endogenous DAMPs and exposure to environmental irritants can activate the NLRP3 inflammasome. Most activators of NLRP3 are produced by cellular stress responses (Baldrighi et al., 2017). The mechanisms by which NLRP3 senses cellular stress and the signaling pathways involved in NLRP3 activation and inflammasome formation are poorly understood. Activation of NLRP3 may involve multiple upstream pathways, but there is no consensus model. Upstream signals of NLRP3 activation are often interrelated and overlapping, including ion internalization and efflux, lysosomal disruption, mitochondrial disorders, metabolic dysfunction, and sphingolipid metabolic changes (Latz et al., 2013).

Radiation can activate NLRP3 inflammasome via multiple mechanisms. Radiation-related potassium ion efflux and calcium flux have been studied extensively (Merrick and Bruce, 1965; Konings, 1981; Franzen et al., 1991, 1993; Todd and Mikkelsen, 1994). Calcium influx into the cytosol can occur through opening of plasma membrane channel or release from the endoplasmic reticulum (ER) intracellular Ca^2+^ pool. Potassium efflux functions as a counter regulator of Ca^2+^ influx. As such, Ca^2+^ influx and K^+^ efflux are often found to be coordinated in NLRP3 activation (Yaron et al., 2015). Adenosine triphosphate is another key signaling molecule that mediates radiation-induced biological effects (Kojima et al., 2017). Stimulation of the P2X purinoceptor 7 (P2X_7_) by ATP induces weak Ca^2+^ and Na^+^ influx, with concurrent K^+^ efflux (Gombault et al., 2012; Karmakar et al., 2016). Low intracellular K^+^ has been shown to activate the NLRP3 inflammasome in THP1 cells and bone-marrow-derived macrophages *in vitro* (Petrilli et al., 2007). Radiation can also directly induce cholesterol biosynthesis (Werner et al., 2019). Longitudinal trends in total serum cholesterol levels of atomic bomb survivors also supported the ability of radiation to induce cholesterol biosynthesis (Wong et al., 1999). Phagocytosis of cholesterol crystals results in lysosomal rupture and release of particles into the cytoplasm. Radiation can directly induce lysosomal destabilization (Persson et al., 2005). Cholesterol crystals and radiation have been shown to induce lysosomal rupture, resulting in NLRP3 activation (Duewell et al., 2010). In addition, cathepsins released from ruptured lysosomes have been shown to play a critical role in NLRP3 activation following radiation treatment (Amaral et al., 2018). However, individual knockouts of cathepsin B, X, L, or S had little effect on NLRP3 activation, which demonstrated that cathepsins may have redundant roles in activation of NLRP3 signaling (Orlowski et al., 2015). Recent studies have suggested that radiation-induced up-regulation of cellular ceramide (Kolesnick and Fuks, 2003; Sharma and Czarnota, 2019) acts as a second messenger in initiation of intrinsic apoptosis. Ceramide plays multiple pathophysiological roles during radiation-induced NLRP3 activation. Ceramide-induced cathepsin D activation has been shown to link TNFα-induced acid sphingomyelinase to Bid-related mitochondrial apoptosis (Heinrich et al., 2004). Recent studies identified plasma membrane ion channels as novel targets of ceramide, such as those responsible for Ca^2+^ influx and K^+^ efflux (Szabo et al., 1996; Lepple-Wienhues et al., 1999; Church et al., 2005). These findings show that many mechanisms of NLRP3 activation involve either Ca^2+^ or K^+^ flux.

Most radiation damage results from water radiolysis, as 80% or more of total cell mass or tissue is comprised of water. Ionizing irradiation-induced water radiolysis generates reactive oxygen species (ROS), the main source of radiation-induced tissue damage. NLPR3 protein contains a highly conserved disulfide bond connecting the PYD domain and the nucleotide-binding site domain that is highly sensitive to altered redox state (Bae and Park, 2011). Radiation-induced mitochondrial dysfunction, and release of mitochondrial ROS and DNA into the cytoplasm, are essential upstream regulators of NLRP3 activation (Chen et al., 2016). Ionizing radiation can also effectively generate ozone, which has been suggested to have a strong association with cardiovascular injury (Srebot et al., 2009; Wang M. et al., 2019). Ozone induced oxidative stress causes inflammasome activation with the release of IL-1 and inflammatory cytokines (Michaudel et al., 2016). ROS may serve as both “kindling” and “bonfire” for NLRP3 inflammasome activation. ROS can function as a redox signaling messenger to trigger NLRP3 inflammasome activation (Abais et al., 2015). When NLRP3 inflammasome is activated, “bonfire” ${\text{O}}_{2}^{∙-}$ and inflammatory cytokines are generated. Excess ROS results in intracellular and extracellular oxidative stress, and damage of nucleotides, lipids, proteins eventually and inevitably. Although there is a plethora of evidence supporting radiation-induced ROS activation of NLRP3 inflammasomes, the exact mechanisms are still largely unknown. Two distinct proteins, thioredoxin-interacting protein (TXNIP) and mitochondrial antiviral signaling protein (MAVS), are demonstrated as possible mediators of ROS to activate NLRP3 inflammasomes (Zhou et al., 2010; Subramanian et al., 2013). Moreover, irradiation damage induces mitosis and pyroptosis, resulting in generation of DAMPs and activation of the NLRP3 inflammasome. Reports have shown persistent expression of NF-κB, which promotes NLRP3 transcription, in patient neck vessels over 10 years post-radiation (Halle et al., 2010). This finding further indicated a possible link between NF-κB-dependent NLRP3 activation and chronic fibrosis.

## The Role of the NLRP3 Inflammasome in Radiation-Induced Cardiovascular Diseases

Recent studies have indicated that upregulation of the NLRP3 inflammasome may play an essential role in RIHD (Wei et al., 2019). NLRP3 inflammasome has been shown as the primary generator responsible for IL-1 production in atherosclerosis and other CVDs (Grebe et al., 2018). Significant increase in secretion of inflammatory cytokines IL-1β and IL-18 was reported in irradiated-animal models (Hong et al., 1999; Ha et al., 2014). IL-1 was suggested to participate in the development of radiation-induced cardiomyopathy (Mezzaroma et al., 2015). Gene expression of IL-1β was significantly elevated during the first month in rat heart after local ionizing irradiation (Kruse et al., 2001). A markedly increased IL-1 expression was observed in irradiated human arteries and veins when compared to non-irradiated (Christersdottir Bjorklund et al., 2013). Increased level of pro-caspase-1 and caspase 1 in human arteries with chronic radiation injury indicated the involvement of NLRP3 inflammasome in RIHD (Christersdottir et al., 2019). However, the outcomes of vascular damage at organ subsites have not been fully characterized, for example, the underlying mechanisms of NLRP3 in radiation-induced CVDs have not yet been established. In this section, we summarized current knowledge of the consequences and molecular mechanisms of RIHD, with a particular focus on the NLRP3 inflammasome in RIHD.

### NLRP3 Inflammasome in Radiation-Induced Atherosclerosis

Activation of the NLRP3 inflammasome has been extensively investigated in macrophages and atheroma cells, including smooth muscle cells (SMC), endothelial cells (ECs), and T cells, each of which play significant roles in onset and development of atherosclerosis (Galkina and Ley, 2009). Recent studies suggested that NLRP3 may have additional functions beyond a regulatory role in inflammasome signaling and the innate immune response (Henao-Mejia et al., 2012). These findings indicated that NLRP3 may be involved in radiation-induced atherosclerosis.

Many endogenous danger signals are present in radiation-induced atherosclerosis, which indicates that the NLRP3 inflammasome may be linked to metabolic disturbances, irradiation damage, and inflammation. Limited studies have evaluated the role of the NLRP3 inflammasome in the pathogenesis of irradiation-induced atherosclerosis, but the role of the NLRP3 inflammasome has been extensively studied in non-irradiation-induced atherosclerosis.

Several epidemiological studies have shown a correlation between aortic NLRP3 expression and CVD (Grebe et al., 2018). Components of the NLRP3 inflammasome are expressed in atheromatous cells (Baldrighi et al., 2017). High aortic expression of NLRP3 has been observed in patients with coronary atherosclerosis. The expression of NLRP3 was correlated with the severity of narrowing or blockage of the coronary artery (Wang et al., 2014). Moreover, aortic NLRP3 expression has been shown to be directly associated with CVD severity and multiple clinical risk factors (Zheng et al., 2013). Analysis of human carotid atherosclerotic plaques showed significantly elevated expression of NLRP3, ASC, caspase-1, IL-1β, and IL-18 compared to those in control mesenteric or iliac arteries (Shi et al., 2015). Furthermore, symptomatic patients with CVD have markedly higher NLRP3 expression than asymptomatic patients. Active atherosclerotic plaques exhibit higher expression of NLRP3 inflammasome components than stable atherosclerotic plaques (Paramel Varghese et al., 2016). In addition, patients with acute coronary syndrome showed significantly increased NLRP3 expression in peripheral blood monocytes, which was directly associated with the severity of the disease (Zhu et al., 2019).

Two murine models developed on the C57BL/6 background, *Apoe*^–/–^ and *Ldlr*^–/–^, have been widely used to investigate the mechanisms of atherogenesis (Emini Veseli et al., 2017). However, these models leverage distinctly different mechanisms to induce diet-induced atherosclerosis (Getz and Reardon, 2016). The importance of activation of the NLRP3 inflammasome in irradiation-induced atherogenesis was first detailed by Duewell et al. (2010), who found that bone marrow transplanted from *Nlpr3*^–/–^, *Asc*^–/–^, or *Il1*α^–/–^/*Il1*β^–/–^ mice into irradiated-*Ldlr*^–/–^ mice resulted in impaired progression of atherogenesis. Hendrikx et al. (2015) recently showed that activation of caspase-1 and caspase-11 were important in diet-induced atherosclerosis using irradiated *Ldlr*^–/–^ mice transplanted with bone marrow from caspase-1 or caspase-11 deficient mice.

Ionizing radiation can also accelerate the development of atherosclerotic lesions in *Apoe*^–/–^ mice (Stewart et al., 2006). Depletion of inflammasome components has been shown to suppress atherogenesis in *Apoe*^–/–^ mice. Zheng et al. (2014) showed that NLRP3 inflammasome activation was involved in atherogenesis by silencing *NLRP3* in *Apoe*^–/–^ mice, which resulted in decreased atherogenesis. Two other groups showed reduced atherogenic plaque progression in *caspase-1*^–/–^/*Apoe*^–/–^ dual knockout mice fed a low cholesterol diet (Gage et al., 2012; Usui et al., 2012). In addition, spontaneous development of atherosclerosis was reduced in chow-fed *caspase-1*^–/–^/*Apoe*^–/–^ mice. Inhibition of IL-18 activity or IL-18 deletion in *Apoe*^–/–^ mice markedly attenuated atherosclerotic plaque progression compared to control mice (Mallat et al., 2001). In addition, *IL-1*β^–/–^/*Apoe*^–/–^ knockout mice showed a 30% reduction in plaque development compared to *Apoe*^–/–^ control mice (Kirii et al., 2003). Another study showed a prominent reduction in plaque size in both *Il1*β^–/–^/*Apoe*^–/–^ and *Il1*α^–/–^/*Apoe*^–/–^ double knockout mice compared to that in *Apoe*^–/–^ control mice. Similar findings were observed in radiation-treated *Apoe*^–/–^ mice transplanted with bone marrow from *Il1*α^–/–^ or *Il1*β^–/–^ mice (Kamari et al., 2011). However, *Nlpr3*^–/–^/*Apoe*^–/–^, *Asc*^–/–^/*Apoe*^–/–^, and *caspase-1*^–/–^/*Apoe*^–/–^ mice did not exhibit decreased progression of atherosclerosis, which indicated that atherosclerosis can proceed independently of NLRP3 inflammasome activation (Menu et al., 2011). Although *Apoe*^–/–^ mice exhibit more rapid and more severe progression of diet-induced atherosclerosis than *Ldlr*^–/–^ mice, spontaneous atherosclerosis, even when fed a normal chow diet, is frequently observed in *Apoe*^–/–^ mice (Getz and Reardon, 2016). The suitability of the *Apoe*^–/–^ mouse model for study of atherogenesis remains controversial. A possible explanation is that compensatory inflammatory signaling may drive progression of atherogenesis at later stages of the disease (Grebe et al., 2018). This hypothesis was supported by findings that both IL-1β and IL-1α signaling are required for atherogenesis (Freigang et al., 2013). IL-1α immediately presents its bioactive form after transcription in a NLRP3 inflammasome-independent manner (Hansson, 2017). Prolonged atherogenic stimulation in *Ldlr*^–/–^ mice transplanted with bone marrow of *Il1*α^–/–^ mice resulted in less significant atherosclerotic lesions than those observed in mice transplanted with bone marrow from *Il1*β^–/–^ mice (Freigang et al., 2013). These findings highlighted the involvement of IL-1α, but not IL-1β, in atherogenic progression following prolonged administration of an atherogenic diet.

Though the long-term adverse effects of radiation on organs, and the involvement of activation of the NLRP3 inflammasome in radiation-induced injury, have been extensively demonstrated (Wei et al., 2019), the underlying mechanisms responsible for RIHD are poorly understood. Whether the NLRP3 inflammasome plays a role in onset or development of radiation-accelerated atherogenesis, or both, has not been determined.

Multiple atherogenic factors, which can be induced by ionizing radiation, have been shown to activate the NLRP3 inflammasome, followed by endothelial damage and subsequent atherogenesis. Activation of the NLPR3 inflammasome has been reported to directly induce endothelial dysfunction (Zhang et al., 2015; Erdei et al., 2018; Koka et al., 2019). A recent study showed that inhibition of radiation-induced NLRP3 inflammasome activation by LGM2605, an antioxidant and free radical scavenger, alleviated vascular damage (Chatterjee et al., 2019). Inhibitors of HMGB1 or caspase-1 significantly ameliorated endothelium-associated vasodilation in poloxamer 407 (P-407)-treated *Nlrp3*^+/+^ mice (Zhang et al., 2015). In addition, activation of endothelial NLRP3 inflammasome was partially correlated with lysosomal disruption in coronary arteritis, which resulted in endothelial dysfunction (Chen et al., 2015). Induction of cold-inducible RNA-binding protein by irradiation causes endothelial dysfunction via activating NLRP3 inflammasome (Yang et al., 2016). Heme, released from damaged RBCs (possibly by radiation under certain conditions), can function as a DAMP to induce IL-1β secretion through NLRP3 activation (Erdei et al., 2018). Heme-induced elevation of IL-1β levels was intensified by LPS priming (Erdei et al., 2018). Acute hypercholesterolemia has been shown to significantly impair endothelium-associated vasodilation in wide-type mice. However, this effect was markedly attenuated in *Nlrp3*^–/–^ mice (Zhang et al., 2015). Li and colleagues recently found that acid sphingomyelinase (ASMase, encoded by gene *Smpd1*) and ceramide-associated membrane raft (MR) signaling platforms were involved in endothelial NLRP3 inflammasome activation and atherogenesis resulting from hypercholesterolemia (Koka et al., 2017). Cholesterol crystal administration significantly promoted NLRP3 inflammasome formation and activation in murine carotid arterial endothelial cells, as evidenced by augmented colocalization of NLRP3 with other inflammasome components and enhanced caspase-1 activity and IL-1β production. These effects were significantly reduced by silencing or deletion of mouse *Smpd1* or treatment with an ASM inhibitor. Neointimal formation that is more severe, significant intimal inflammasome formation, and increased IL-1β expression were associated with elevated NLRP3 in *Smpd1*^+/+^ mice compared with those in *Smpd1*-deficient mice. These data showed that ASMase and ceramide-related MR clustering are critical for activation of inflammasomes and subsequent endothelial dysfunction in the carotid arteries, resulting in progression of atherosclerosis (Koka et al., 2017).

Radiation can directly damage cardiovascular endothelial cells through multiple mechanisms, which can further enhance the pathophysiological effects of the NLRP3 inflammasome. Single and fractionated ionizing radiation significantly affect vasculature by altering endothelial expression of connexins and increase release of ATP (Ramadan et al., 2019). Conventional fractionated ionizing radiation induces mitotic catastrophe (mitotic cell death distinct from apoptosis) in ECs (Galluzzi et al., 2018). Radiation (≥10 Gy) can induce cell cycle arrest and Bax-mediated apoptosis of endothelial progenitor cells (EPCs), and cause cellular senescence in well-differentiated ECs similar to that observed in premature atherosclerosis (Venkatesulu et al., 2018). Radiation-induced cell death, which is characterized by mitotic catastrophe, apoptosis, and senescence, could stimulate an acute reduction in capillary density or induce chronic inflammation, resulting in disruption of vascular homeostasis (Venkatesulu et al., 2018).

Radiation-related DNA damage-induced EC senescence often results in secretion of cytokines, proteins, and extracellular vesicles (Alique et al., 2018). The senescence-related secretory phenotype of ECs results in dysfunction of adjacent cells or chronic inflammation. A study showed that irradiation increased the expression of the aging-related atherosclerotic senescence marker CD44 (Mun and Boo, 2010) in human coronary ECs and increased monocyte adhesion to ECs (Lowe and Raj, 2014). Radiation can also activate NEMO-dependent NF-κB signaling, which is involved in transcription and upregulation of NLRP3 (Wang S. et al., 2018), cytokines (Meeren et al., 1997), and adhesion molecules (Linard et al., 2004). Furthermore, irradiation also activates TNFα, resulting in ceramide generation via hydrolysis of sphingomyelin. Ceramides can induce the expression or activation of NF-κB, an important proinflammatory transcription factor (Kitajima et al., 1996; Gill and Windebank, 2000), Moreover, ceramides can directly trigger NLRP3 inflammasome activation (Vandanmagsar et al., 2011; Kolliputi et al., 2012; Scheiblich et al., 2017). In addition, radiation disrupts inflammatory homeostasis in the vascular microenvironment by increasing the expression of selectins, integrins, and adhesion molecules such as intercellular adhesion molecule 1 (ICAM-1) and vascular cell adhesion molecule 1 (VCAM-1) (Handschel et al., 1999; Prabhakarpandian et al., 2001). However, the effect of radiation on initiation of NF-κB/NLRP3 signaling, upregulation of adhesion molecules, and recruitment of monocytes/macrophages depends on the types and fractions of radiation, and the types of endothelial cell exposed to radiation (Mavragani et al., 2016). Irradiation-induced EC dysfunction may occur in NLRP3 inflammasome-dependent and -independent manners.

Vascular smooth muscle cells (VSMCs), which are major components of medium and large arteries, participate in arterial wall remodeling and atherogenesis throughout all stages of atherosclerosis (Hu et al., 2019). Although mechanisms of atherogenesis remain poorly understood, studies have shown that crosstalk between inflammatory cells and ECs and VSMCs contributes to CVD progression. Some of these interactions accelerate the genesis and stability of atherosclerotic plaques (Li et al., 2018). Tracing of SMC lineage in *Apoe*^–/–^ mice fed a long-term western-diet has contributed to understanding of the role of VSMC in atherosclerosis (Shankman et al., 2015). Vascular smooth muscle cells in atherosclerotic lesions have been shown to be derived from a subset of medial Myh11^+^ VSMCs that propagate the lesions (Chappell et al., 2016). Most VSMCs within atherosclerotic sites lack characteristic SMC marker expression profiles, but they express markers of mesenchymal stem cells, myofibroblast-like cells, or macrophages (Bennett et al., 2016). Milliat et al. (2006) demonstrated the role of proliferation, migration, and fibrogenic phenotype transition of VSMCs in irradiation-induced vascular damage. Co-culture of VSMCs with irradiated ECs resulted in significantly increased proliferation and migration. Further study showed that TGF-β/Smad3 signaling played a role in irradiated EC-induced fibroblastic phenotype transition of VSMCs. However, the role of the NLPR3 inflammasome in VSMC-mediated atherosclerosis has not been characterized. Wang and colleagues found that NLRP3 inflammasome activation promoted foam cell formation in human VSMCs and induced atherogenesis in *Apoe*^–/–^ mice via release of HMGB1 (Wang R. et al., 2018). Moreover, NLRP3 inflammasome activation has been shown to induce VSMC synthetic phenotype transition and proliferation (Sun et al., 2017). Deficiency of NLRP3 has been shown to reduce Ang II-induced NLRP3 inflammasome activation, synthetic phenotypic transition, and proliferation in murine VSMCs (Ren et al., 2017). However, Duewell et al. (2010) found that lethal irradiation (12 Gy) of *Apoe*^–/–^ mice resulted in loss of VSMC accumulation in the brachiocephalic and carotid artery, but VSMC levels in the aortic root and in abdominal aortic lesions were normal (Newman et al., 2018). These studies demonstrated differences in the sensitivity to radiation of VSMCs in different vascular beds.

Priming and activation signals are required for NLRP3 inflammasome activation in macrophages. This two-step mechanism tightly regulates activation of NLRP3 inflammasome in macrophages. Nucleotide-binding domain and leucine-rich-repeat-containing family pyrin 3 is easily activated by a variety of DAMPs generated by ionizing radiation. Radiation can induce inflammatory cytokine production in macrophages (Teresa Pinto et al., 2016) and can trigger NLRP3 inflammasome activation, resulting in macrophages pyroptosis (Liu et al., 2017). Pyroptosis is a rapid and highly inflammatory form of programmed cell death that can be initiated by DAMP-activated NLRP3 inflammasome (Guo et al., 2015). Moreover, NLRP3 inflammasome activation has been shown to augment lipid-deposition and macrophage migration (Li et al., 2014). Macrophage recruitment is the primary response to radiation-induced damage (Ahn et al., 2010), and macrophage polarization is affected during the early and late stages of radiation-induced tissue damage (Duru et al., 2016; Meziani et al., 2018). Radiation promotes pro-inflammatory M1 macrophage polarization, which results in enhanced production of M1 cytokines such as TNF-α, interleukins, and IFN-γ (Farooque et al., 2016). Mature M1 macrophages accelerate extracellular matrix breakdown, tissue damage, and fibrosis (Braga et al., 2015). In fact, ionizing radiation can activate NLRP3 inflammasome in multiple immune cells, including T and B lymphocytes (Stoecklein et al., 2015). Both myeloid and adaptive response are involved in atherogenesis (Wolf and Ley, 2019).

### NLRP3 Inflammasome Regulation of Radiation-Induced Cardiovascular Fibrosis

The endpoint of RIHD is cardiovascular fibrosis, which is a severe complication associated with a high rate of mortality. Radiation-induced fibrotic diseases of the cardiovascular system include radiation-related coronary artery disease with manifest fibrous tissue, myocardial fibrosis, cardiomyopathy, valvular fibrosis, and fibrosis of the pericardium. Vascular endothelial damage is widely considered to be the primary pathogenic signal that precedes fibrosis (Yarnold and Brotons, 2010).

Radiation damage induces early and delayed cardiac tissue injury. Ionizing radiation induces early endothelial damage, which results in vasodilation and increased blood vessel permeability (Soloviev et al., 2003). Injured ECs secrete inflammatory cytokines (TNFα, IL-1, IL-6, and IL-8), resulting in upregulation of adhesion molecules and activation of the inflammatory response. Inflammatory cells recruited to lesions produce profibrotic cytokines including transforming growth factor-beta (TGF-β), platelet-derived growth factor (PDGF), basic fibroblast growth factor (bFGF), and connective tissue growth factor (Cuomo et al., 2016). Radiation-induced cardiovascular injury triggers a complex cascade of cellular and molecular responses that result in tissue fibrosis. Fibrogenesis consists of four major phases: (1) The initiation stage is driven by primary vascular endothelial injury. The central event during this phase is recruitment of inflammatory cells, which produce a number of mediators, cytokines, and other factors that strengthen the inflammatory response; (2) Activation of fibrogenic effector cells, include fibroblasts, fibrocytes, tissue-specific pericytes, and myofibroblasts. These types of cells can proliferate and differentiation into mature myofibroblasts in response to injury; (3) Elaboration of the extracellular matrix occurs when effector cells produce a large amount of extracellular matrix proteins, primarily in an autocrine manner. In addition, cell–cell interactions lead to further activation of effector cells, resulting in a positive feedback loop. This process is driven by TGF-β and PDGF; (4) The final stage is fibrosis, which results in organ dysfunction and failure (Rockey et al., 2015).

**FIGURE 1:**
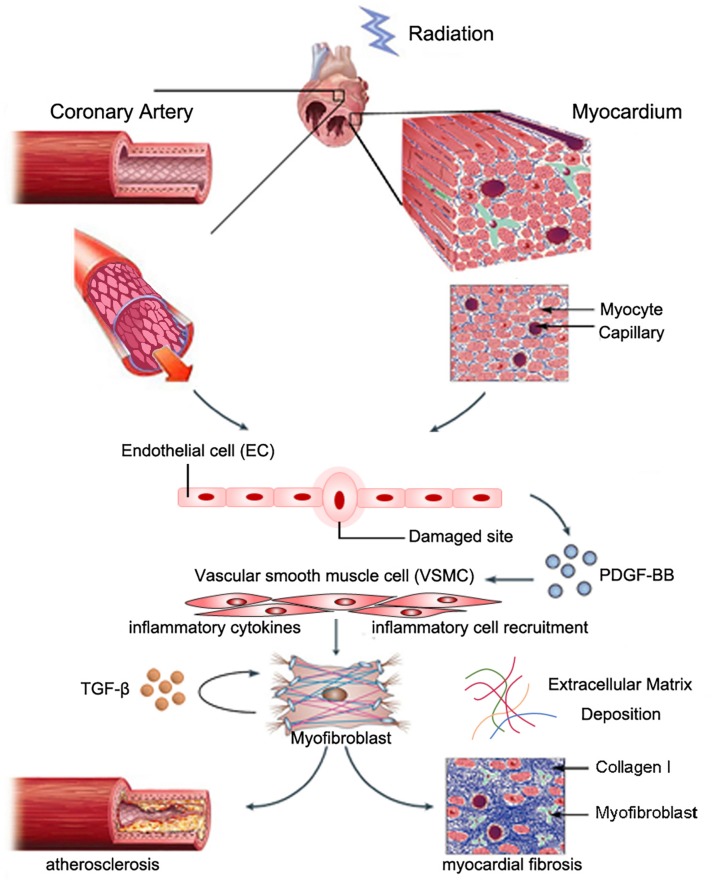
Possible role of VSMCs in radiation-induced cardiac fibrosis. Partially reproduced with permission from Weintraub et al. (2010).

Myocardial fibrosis commonly features reactive fibrosis and replacement fibrosis (Travers et al., 2016). Reactive fibrosis often occurs in the perivascular space, and replacement fibrosis occurs at locations where myocytes are lost. Radiation-induced myocardial fibrosis may be induced by cardiac fibroblasts, which are the most abundant non-myocyte cell type in the myocardium (Krenning et al., 2010). A recent study indicated that NLRP3 inflammasome in fibroblast linked inflammation with tissue injury (Ershaid et al., 2019). Cardiac fibroblasts might originate from myocardial mesenchymal cells, bone marrow-derived circulating fibroblasts, and epithelial/endothelial–mesenchymal cell transition (EndoMT/EMT) (Moore-Morris et al., 2014). EndoMT is a transition process where ECs lose their endothelial characteristic features and obtain mesenchymal properties. Mounting evidence indicated that EndoMT contributed to cardiac fibrosis (Kovacic et al., 2019). Nearly one-third of all cardiac fibroblasts originated from ECs (Zeisberg et al., 2007). A recent study showed that NLRP3 inflammasome activation during the EMT linked inflammatory injury and endothelial dysfunction (Cho et al., 2018). In addition, studies have shown that myofibroblasts can also originate from resident VSMCs during atherogenesis, as evidenced by expression of smooth muscle myosin heavy chain (SMMHC) and alpha-smooth actin (αSMA). Skin biopsy samples from patients who underwent breast radiotherapy showed collagen accumulation and fibroblast expression of αSMA, which indicated a myofibroblast phenotype. Benderitter and colleagues reported prominent ECM deregulation and VSMC proliferation in hypertrophic blood vessels in patients who received preoperational radiotherapy, as evidenced by the presence of αSMA-expressing cells in the condensed collagen matrix region of intimal hyperplasia. *In vitro* co-culture experiments showed that VSMCs co-cultured with irradiated human endothelial cells underwent myofibroblastic transition (Milliat et al., 2006). Zhang and colleagues recently reported that PDGF-BB stimulation of *Spmd1*-deficient VSMCs induced differentiation to a myofibroblast-like phenotype, as evidenced by marked upregulation of fibroblast-specific protein (FSP-1) expression, massive collagen type I deposition, increased IL-6 release, and upregulation of adhesion molecule expression (Zhang et al., 2018). The underlying mechanism of these effects may be due to prolonged Akt activation, and p62/SQSTM1-dependent NRF2 upregulation and nuclear translocation. These findings suggested that myofibroblasts originated from resident VSMCs (Figure 1).

The NLRP3 inflammasome is also involved in regulation of the expression of profibrotic cytokines (e.g., TGF-β, PDGF, and IL-6) (Anders et al., 2018). Wang et al. (2013) showed that NLRP3 inflammasome activation augmented TGF-β signaling in the kidney epithelium. TGF-β1-induced EMT, and induction of MMP-9 and αSMA were significantly impaired in *Nlrp3*-deficient murine renal tubular epithelial cells (TECs). Mitochondrial NLRP3 protein has been shown to directly induce ROS production, resulting in increased TGF-β/Smad signaling and fibrosis without inflammasome formation (Bracey et al., 2014). The crosstalk between inflammasome-independent NLRP3 and TGFR signaling suggests the essential role of NLRP3 in myofibroblast differentiation and ECM accumulation (Anders et al., 2018). A recent study indicated that triptolide (TP) attenuated cardiac fibrosis by interrupting NLRP3-TGFβ1-Smad signaling (Pan et al., 2019). Coriolus versicolor (CV) was also shown to alleviate cardiac fibrosis through suppression of the TGF-β/Smad pathway and NLRP inflammasome activation (Wang Y. et al., 2019). Similarly, NLRP3 deficiency resulted in reduced TGF-β- and PDGF-BB-induced proliferation of *Nlrp3*-deficient renal fibroblasts (Anders et al., 2018). Kawaguchi et al. (2011) showed that hypoxia-reoxygenation-induced ROS production and K^+^ efflux stimulated inflammasome activation in cardiac fibroblasts, but not cardiomyocytes. Radiation has been shown to induce robust production of ROS, which results in differentiation of CD4^+^ T cells into Th2 lymphocytes that secrete potent profibrotic cytokines such as IL-4 and IL-13 (Besnard et al., 2011; Braga et al., 2019). Nucleotide-binding domain and leucine-rich-repeat-containing family pyrin 3 can directly promote Th2 programming without inflammasome formation (Bruchard et al., 2015), NLRP3 can also modulate M2 macrophage polarization via upregulation of IL-4 (Liu Y. et al., 2018). Taken together, these findings indicate that NLRP3 inflammasome exhibits a complex function in radiation-induced cardiovascular fibrosis.

## Therapeutic Targeting of NLRP3 Against Rihd

Characterization of the underlying mechanisms of activation of the NLRP3 inflammasome may allow for development of effective inhibitors or regulators as therapeutic agents for RIHD. A number of candidate NLRP3 inhibitors have exhibited promising therapeutic effects against inflammatory disorders. These inhibitors primarily target NLRP3, other inflammasome components, or related signaling events.

Three therapeutics agents against IL-1/IL-1R have been approved by the US FDA for treatment of multiple inflammatory diseases: anakinra (recombinant antagonist against the IL-1 receptor), rilonacept (decoy receptor that binds IL-1β), and as canakinumab (a specific antibody against IL-1β) (Dinarello et al., 2012). Two other inhibitory antibodies, MAPp1 for IL-1α and GSK1070806 for IL-18, are under development (Swanson et al., 2019). The success of the Canakinumab Anti-inflammatory Thrombosis Outcomes Study (CANTOS) demonstrated the clinical translational potential of inhibitors against NLRP3-driven immunopathological diseases. This study evaluated the impact of canakinumab on occurrence and recurrence of CVDs (e.g., MI, stroke). Standard canakinumab administration resulted in a 15% decrease in MI and cardiovascular mortality compared to placebo. The CANTOS trial was the first study to demonstrate the translational significance of the proatherogenic effect of IL-1β, as evidenced by the finding that clinical outcomes of CVD were improved by interfering with IL-1β production or signaling (Ridker et al., 2012). A recent gene expression analysis of both irradiated and non-irradiated human blood vessels from the Biobank of Radiated Tissues at Karolinska (BiRKa) was conducted, suggesting the involvement of NLRP3-inflammasome and potential treatment using IL-1 inhibitors. According to their findings, treatment with anakinra in irradiated *Apoe*^–/–^ mice significantly alleviated arterial inflammation (Christersdottir et al., 2019).

Development of oral small molecules specific for NLRP3 inflammasome inhibition might be a more promising, less invasive, and more cost-effective strategy (Zahid et al., 2019). Among the inhibitors summarized in Table 1, compound MCC950 is the most promising NLRP3 inhibitor candidate because it selectively suppresses NLRP3 inflammasome signaling in human and mouse macrophages. In addition, MCC950 does not affect non-NLRP3 inflammasomes, TLR-mediated priming signals, or IL-1α releasing. MCC950 exhibits striking therapeutic effects against a variety of preclinical immunopathological models such as atherosclerosis, cardiac arrhythmia, MI, and colitis (Liston and Masters, 2017; Swanson et al., 2019). Another NLRP3 specific inhibitor, 16673-34-0 exerted protective effects against myocardial ischemia-reperfusion injury and acute peritonitis (Lipinski and Frias, 2014). Pharmacological interventions targeting alternative inflammasome constituents are being actively investigated (Zahid et al., 2019). Of note, only a small number of inhibitory therapeutics that target NLRP3 or its downstream effectors and pathways show potent efficacy for treatment of NLRP3-associated diseases. Other inhibitors such as AZD9059 and GSK1482169 for P2 × 7, and VX VX-740 and VX-756 for caspase-1 also did not induce therapeutic effects against NLRP3-related diseases due to ineffectiveness or severe hepatic toxicity (Ozaki et al., 2015). Interestingly, non-specific pharmacological interventions can also modulate NLRP3 inflammasome activity. Atorvastatin was found to effectively suppress NLRP3 inflammasome level in CAD (Satoh et al., 2014). Certain antidiabetic medicines such as biguanides, thiazolidinediones, and inhibitors of dipeptidyl peptidase 4 have the potential to regulate the activation of NLRP3 inflammasome (Zhang and Wang, 2020). An antioxidant, melatonin, could also function as an anti-inflammatory agent by inhibiting the activity of NLRP3 inflammasome (Garcia et al., 2015).

**TABLE 1:** Inhibitors against NLRP3 inflammasome.

Target specificity		Inhibitor	Diseases	Clinical status	References
	NLRP3	MCC950	Inflammatory diseases	Phase II	Coll et al., 2015
	NLRP3	Tranilast (MK-341)	Inflammatory diseases	Approved	Huang et al., 2018
	NLRP3	OLT1177	Inflammatory diseases	Phase II	Marchetti et al., 2018
	NLRP3	CY-09	Inflammatory diseases	Preclinical	Jiang et al., 2017
	NLRP3	JC171	Inflammatory diseases	Preclinical	Guo et al., 2017
	NLRP3	JC124	Traumatic neuroinflammatory response	Preclinical	Kuwar et al., 2019
	NLRP3	INF39	Inflammatory diseases	Preclinical	Cocco et al., 2017
	NLRP3	NBCs	Inflammatory diseases	Preclinical	Baldwin et al., 2017
	NLRP3	Apigenin	Inflammatory diseases	Preclinical	Lim et al., 2018
	NLRP1, NLRP3, NK-κB, IKKβ	Parthenolide	Inflammatory diseases, Cystic fibrosis	Preclinical	Juliana et al., 2010
	NLRP3	ILG	Obesity, hypercholesterolemia, and insulin resistance	Preclinical	Honda et al., 2014
	NLRP3	Fc11a-2	Inflammatory diseases	Preclinical	Liu et al., 2013
NLRP3	NLRP3	BOT-4-one	Inflammatory diseases	Preclinical	Shim et al., 2017
	NLRP3	Glyburide	Inflammatory diseases	Preclinical	Lamkanfi et al., 2009
	NLRP3	MNS	Inflammatory diseases	Preclinical	He et al., 2014
	NLRP3	Flufenamic acid	Alzheimer’s disease	Preclinical	Daniels et al., 2016
	NLRP3	16673-34-0	Acute myocardial infarction	Preclinical	Marchetti et al., 2014
	NLRP3	Oridonin	Inflammatory diseases	Preclinical	He et al., 2018
	NLRP3	Kynurenic acid	Stress-related colonic disorder	Preclinical	Zheng et al., 2019
	NLRP3	Formononetin	Inflammatory diseases	Preclinical	Wu D. et al., 2018
	NLRP3	Triptolide	Cardioprotective	Preclinical	Li et al., 2017
	NLRP3	Andrographolide	Inflammatory diseases	Preclinical	Guo et al., 2014
	NLRP3	Celastrol	Inflammatory diseases	Preclinical	Xin et al., 2017
	NLRP3	BHB	Inflammatory diseases	Preclinical	Youm et al., 2015
Caspase-1	Caspase-1	VX-740	Inflammatory diseases	Preclinical	Rudolphi et al., 2003
	Caspase-1	VX-765	Inflammatory diseases	Preclinical	Wannamaker et al., 2007
	IL-1R1	Anakinra	Cystic fibrosis	Approved	Iannitti et al., 2016
	IL-1β	Canakinumab	Cardiovascular events	Approved	Shah et al., 2018
	IL-1β	Gevokizumab	Cardiac remodeling and coronary dysfunction	Preclinical	Harouki et al., 2017
	IL-1β	Pralnacasan	Inflammatory diseases	Preclinical	Rudolphi et al., 2003
Cytokines	IL-1β	Curcumin	Inflammatory diseases	Preclinical	Gong et al., 2018
	IL-1βR	Rilonacept	CAPS	Approved	Gillespie et al., 2010
	IL-1α	Xilonix	Femoral artery restenosis	Phase II	El Sayed et al., 2016
	IL-4/IL-13	SAR156597	IPF	Phase II	Raghu et al., 2018
	TGF-β	SHP-627	Cardiac fibrosis	Preclinical	Zhang et al., 2012
	PDGFR	Imatinib	NSF, IPF	Phase II	Daniels et al., 2010
	Endothelin-1 receptor	Macitentan	Heart failure	Phase II	NCT03153111
	Angiotensin II	Losartan	Cardiac fibrosis	Preclinical	Gay-Jordi et al., 2013
Related pathways	Relaxin receptor	Serelaxin	Cardiac fibrosis	Preclinical	Wu X. P. et al., 2018
	IKK	IMD-1041	Cardiac fibrosis	Preclinical	Tanaka et al., 2012
	IKK	Bardoxolone methyl	Pulmonary hypertension	Phase II	Chin et al., 2018
	NF-κB	BAY 11-7082	Inflammatory diseases	Preclinical	Irrera et al., 2017
	NK-κB	Baicalein	Renal fibrosis	Preclinical	Zhao et al., 2016
	NF-κB	Wogonoside	Inflammatory diseases	Preclinical	Sun et al., 2015
	JNK	Tanzisertib (CC930)	IPF	Phase II	NCT01203943
	TLR-4	Alpinetin	Inflammatory diseases	Preclinical	He X. et al., 2016
	MAPK	MMI-0100	Cardiac fibrosis	Preclinical	Meng et al., 2017
	Peroxiredoxin 1	AI-44	Inflammatory diseases	Preclinical	Liu W. et al., 2018
	AhR/Nrf2/NQO1	Cardamonin	Inflammatory diseases	Preclinical	Wang K. et al., 2018
	AMPK/ULK1/p62	Ginsenoside Rd	Inflammatory diseases	Preclinical	Liu C. et al., 2018
	Cathepsin B	VBY-376	Liver fibrosis	Preclinical	Wree et al., 2016
	Smad2/4	Pentoxifylline	Cardiac fibrosis	Preclinical	Zhang et al., 2016
	NOX4	GKT-137831	Cardiac fibrosis	Preclinical	Zhao et al., 2015
	ROS	*N*-acetylcysteine	Cardiac fibrosis	Preclinical	Giam et al., 2016
	ROS	Levornidazole	Inflammatory diseases	Preclinical	Wang et al., 2015
	ROS	Mitoquinone	Cardiac fibrosis	Preclinical	Goh et al., 2019
	ROS	LGM2605	Radiation damage	Preclinical	Chatterjee et al., 2019

## Conclusion and Perspectives

**FIGURE 2:**
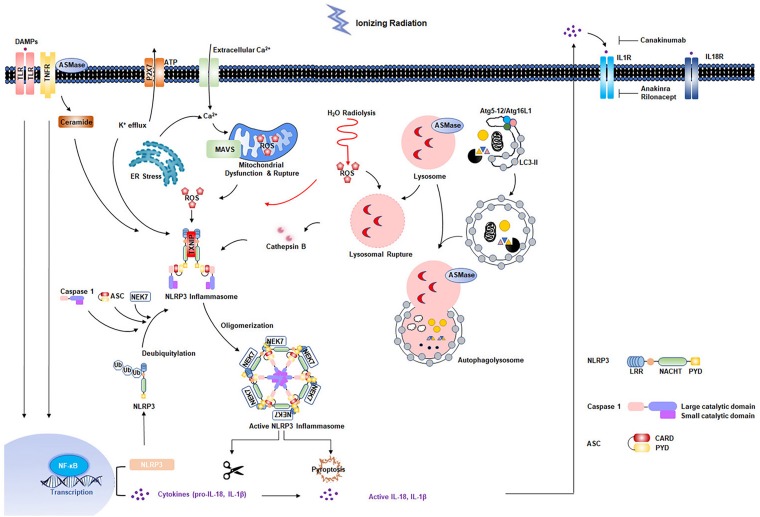
Multiple effects of radiation on NLRP3 inflammasome activation. Radiation-induced NF-κB activation is responsible for the upregulation of NLRP3 and pro-interleukin-1β. Second signals come from multiple pathways: water radiolysis, K^+^ efflux, endoplasmic reticulum (ER) stress, mitochondrial dysfunction, ceramide pathway, and lysosomal rupture pathways, most of which appear to converge in the production of reactive oxygen species (ROS). The primary and secondary signals triggers the NLRP3 inflammasome activation, resulting in the maturation of caspase 1 and IL-1β. Two evolutionarily conserved degradation pathways, ubiquitin-proteasome and autophagy, negatively regulate NLRP3 activation.

Radiation-induced cardiovascular injury has received increased attention due to the growing population of cancer survivors exposed to chest radiation. Although great improvements have been made in radiotherapy techniques, asymptomatic patients are at increased risk for development of RIHD. Exposure to low-dose irradiation may result in adverse cardiovascular effects. Studies have shown that acute and chronic inflammatory responses are essential in development of RIHD. Increasing evidence has indicated that the NLRP3 inflammasome is involved in radiation damage. In addition, multiple cellular effects of radiation, such as ROS production and generation of DAMPs resulting from cell death, can further strengthen NLRP3 inflammasome activation (Figure 2). However, the mechanisms by which the NLRP3 inflammasome functions in RIHD requires further characterization. In addition, previous studies showed that two evolutionarily conserved degradation pathways, ubiquitin-proteasome and autophagy, negatively regulate NLRP3 inflammasome activation (Song et al., 2016; Zhong et al., 2016). Small molecule has been developed to inhibit NLRP3 inflammasome activity by interplaying between ubiquitination and autophagy (Han et al., 2019). MicroRNA was also identified to respond to chronic inflammation in RIHD (Eken et al., 2019). Future translational researches are needed to determine whether NLRP3 inflammasome inhibition and whether these pharmacological candidate inhibitors mentioned above are promising to prevent radiation-induced cardiovascular injury, inflammation, and late fibrosis. Additionally, it seems to be rational to start with an early and short-term IL-1β blockage treatment directly after radiotherapy exposure. It is based on the concept that the intervention might not work effectively in already established RIHD, where the chronic inflammatory response is advanced (Christersdottir et al., 2019). Further studies are required to evaluate the optimal timing and treatment-duration of NLRP3 inflammasome signaling inhibition to prevent RIHD. Experimental animal models for RIHD are also needed to be optimized to accelerate the investigation of the underlying mechanism and development of novel treatment strategies.

## Author Contributions

PZ conceived of the topic for this review. All the authors listed made substantial and intellectual contributions to the work.

## Conflict of Interest

The authors declare that the research was conducted in the absence of any commercial or financial relationships that could be construed as a potential conflict of interest.
